# Genetic variation in brown trout *Salmo trutta* across the Danube, Rhine, and Elbe headwaters: a failure of the phylogeographic paradigm?

**DOI:** 10.1186/1471-2148-13-176

**Published:** 2013-08-26

**Authors:** Estelle Lerceteau-Köhler, Ulrich Schliewen, Theodora Kopun, Steven Weiss

**Affiliations:** 1 Institute of Zoology, Karl-Franzens University Graz, Universitätsplatz 2, A-8010 Graz Austria; 2 Department of Ichthyology, Bavarian State Collection of Zoology (ZSM), Münchhausenstr. 21, D-81247, München Germany; 3 Department of Plant Biology and Forest Genetics,Uppsala BioCenter, Swedish University of Agricultural Sciences (SLU), Box 7080, S-750 07 Uppsala Sweden; 4 Institute of Plant Sciences, Karl-Franzens University Graz, Schubertstraße 51, A-8010 Graz Austria

**Keywords:** Paleo-hydrology, Phylogeography, Alpine, Austria, Bavaria, mtDNA, Microsatellites, LDH-C1, Stocking, Conservation

## Abstract

**Background:**

Brown trout *Salmo trutta* have been described in terms of five major mtDNA lineages, four of which correspond to major ocean basins, and one, according to some authors, to a distinct taxon, marbled trout *Salmo marmoratus*. The Atlantic and Danubian lineages of brown trout meet in a poorly documented contact zone in Central Europe. The natural versus human mediated origin of the Atlantic lineage in the upper Danube is a question of both theoretical and practical importance with respect to conservation management. We provide a comprehensive population genetic analysis of brown trout in the region with the aim of evaluating the geographic distribution and genetic integrity of these two lineages in and around their contact zone.

**Results:**

Genetic screening of 114 populations of brown trout across the Danube/Rhine/Elbe catchments revealed a counter-intuitive phylogeographic structure with near fixation of the Atlantic lineage in the sampled portions of the Bavarian Danube. Along the Austrian Danube, phylogeographic informative markers revealed increasing percentages of Danube-specific alleles with downstream distance. Pure Danube lineage populations were restricted to peri-alpine isolates within previously glaciated regions. Both empirical data and simulated hybrid comparisons support that trout in non-glaciated regions north and northeast of the Alps have an admixed origin largely based on natural colonization. In contrast, the presence of Atlantic basin alleles south and southeast of the Alps stems from hatchery introductions and subsequent introgression. Despite extensive stocking of the Atlantic lineage, little evidence of first generation stocked fish or F_1_ hybrids were found implying that admixture has been established over time.

**Conclusions:**

A purely phylogeographic paradigm fails to describe the distribution of genetic lineages of *Salmo* in Central Europe. The distribution pattern of the Atlantic and Danube lineages is extremely difficult to explain without invoking very strong biological mechanisms.

The peri-alpine distribution of relict populations of pure Danubian lineage brown trout implies that they colonized headwater river courses post-glacially ahead of the expansion of the Atlantic lineage. The recognition of natural as opposed to anthropogenic introgression of the Atlantic lineage into Danubian gene pools is of fundamental importance to management strategies.

## Background

An overwhelming majority of species in nature exhibit some level of phylogeographic structure and this structure is very often shown to correspond to major landscape features born out of paleo-environmental processes. The phylogeographic revolution [1] has promoted a paradigm, especially in Europe, whereby mountain ranges, peninsulas, and for freshwater organisms, river catchments are *a priori* landscape units expected to correspond to some level of genetic subdivision within a species. To the extent that studies support these expectations, the evolutionary significance of this non-biological source of genetic structure remains controversial [2]. Moreover, broad-scale phylogeographic studies of European fishes almost invariable invoke complex within-drainage or between drainage lineage structure, which is presumed to be the result of paleo-hydrological re-arrangements of river networks or for some managed species, the result of human-mediated transport and release. Several studies support a clear distinction between genetic lineages of cold-tolerant fishes across adjacent headwater regions of the Rhine and Danube catchments such as for *Telestes souffia*[3] and European grayling *Thymallus thymallus*[4], but a comparably low level of distinction for *Cottus gobio*[5]. In contrast, lineage sharing between the Danube and Rhine catchments presumably based on natural colonization patterns has been demonstrated in European perch *Perca fluviatilis*[6] and European chub *Leuciscus cephalus*[7] and between the Danube and Vistula drainages for *Barbus carpathicus*[8]. The pre-molecular view of the Ponto-Caspian (including the Danube) basin in aquatic zoogeography was that it served as the major refuge for the post-glacial re-colonization of Central and Northern Europe [9]. While molecular studies highlight the evolutionary importance of the Ponto-Caspian basin in terms of genetic diversity, there is often little evidence or a lack of clarity concerning its role as a source of post-glacial expansion for specific species. None of the above studies were explicitly aimed at investigating the Danube/Rhine/Elbe headwater region with respect to these considerations. Moreover, the population-level knowledge base of the investigated species does not approach that of the brown trout.

The brown trout was one of the first non-model vertebrates to attract the attention of population geneticists in Europe [10-12] and subsequently one of the first organisms to be analyzed with molecular markers for a pan-European phylogeographic structure [13]. Five major mtDNA lineages of the species were identified corresponding to four major catchment areas (Danube, Atlantic, Mediterranean & Adriatic) with the fifth lineage associated with the marbled phenotype found in the Adriatic basin. This structure has proliferated in the literature as the “five major evolutionary lineages” of the species [14]. Subsequent studies based on intensive sampling deliver a more complicated perspective than that of major mtDNA lineages associated with major river basins (e.g. [15-17]). An ancient split within the Atlantic lineage was reported [18] and described as a sixth (Duero) major mtDNA lineage [19]. The allopatric origin of the Mediterranean lineage was questioned [15], and the “Adriatic” lineage was considered a misnomer [16] as it is extensively distributed from Iberia to Turkey, with no evidence that its origin is in the Adriatic. Although the marbled trout phenotype is limited to portions of Adriatic drainage (Po drainage in Italy, Slovenia, Bosnia-Herzegovina & Montenegro), the so-called marble trout mtDNA lineage has been found in Albania [20], central Italy [21], Greece [22] and Corsica (Snoj A. personal communication, unpublished data). Thus, the mtDNA lineage appears to have an independent origin with respect to the taxon’s phenotypic divergence.

In Central Europe, the notion of a Danube lineage of brown trout has been well-established and unquestioned within management and conservation circles. The lineage, however, exhibits extensive life-history diversity and stretches across the entire Ponto-Caspian basin, exhibiting its deepest split across the Tigris-Euphrates catchment [23], leaving some uncertainty about where the lineage actually arose as well as its meaning related to evolutionary or taxonomic debate. Recently, populations of the upper Danube basin have been assigned a new name *Salmo labrax*[24], although it is unclear what the distribution of this taxon should be, or on what basis if any it should be genetically delineated. Weiss et al. [25] reported on the extensive sympatric occurrence of Atlantic and Danube lineage mtDNA in Austrian and Bavarian streams of the upper Danube, and argued that although introduced hatchery strains may account for much of this pattern, the statistical distribution of Atlantic lineage mtDNA suggests a degree of natural occurrence on the north slopes of the Alps. Watershed patterns of alleles at the LDH-C1 locus reflected some known river capture events across adjoining headwater tributaries of the Rhine and Danube in Baden-Württemberg [26]. A review of molecular genetic data including extensive allozyme studies [27] concluded that there is no modern genetic signal of brown trout gene flow from the Ponto-Caspian Basin into north draining river systems of Central Europe (e.g. Rhine & Elbe), despite the generally accepted zoogeographic model of this basin serving northern post-glacial expansion and the fact that the Danube basin has repeatedly lost (through river capture) area to the north-flowing Rhine and Elbe systems over the last few ice ages [28]. Nonetheless, no study to date has systematically evaluated the genetic structure of brown trout across the Danube/Rhine/Elbe interface.

**Figure 1:**
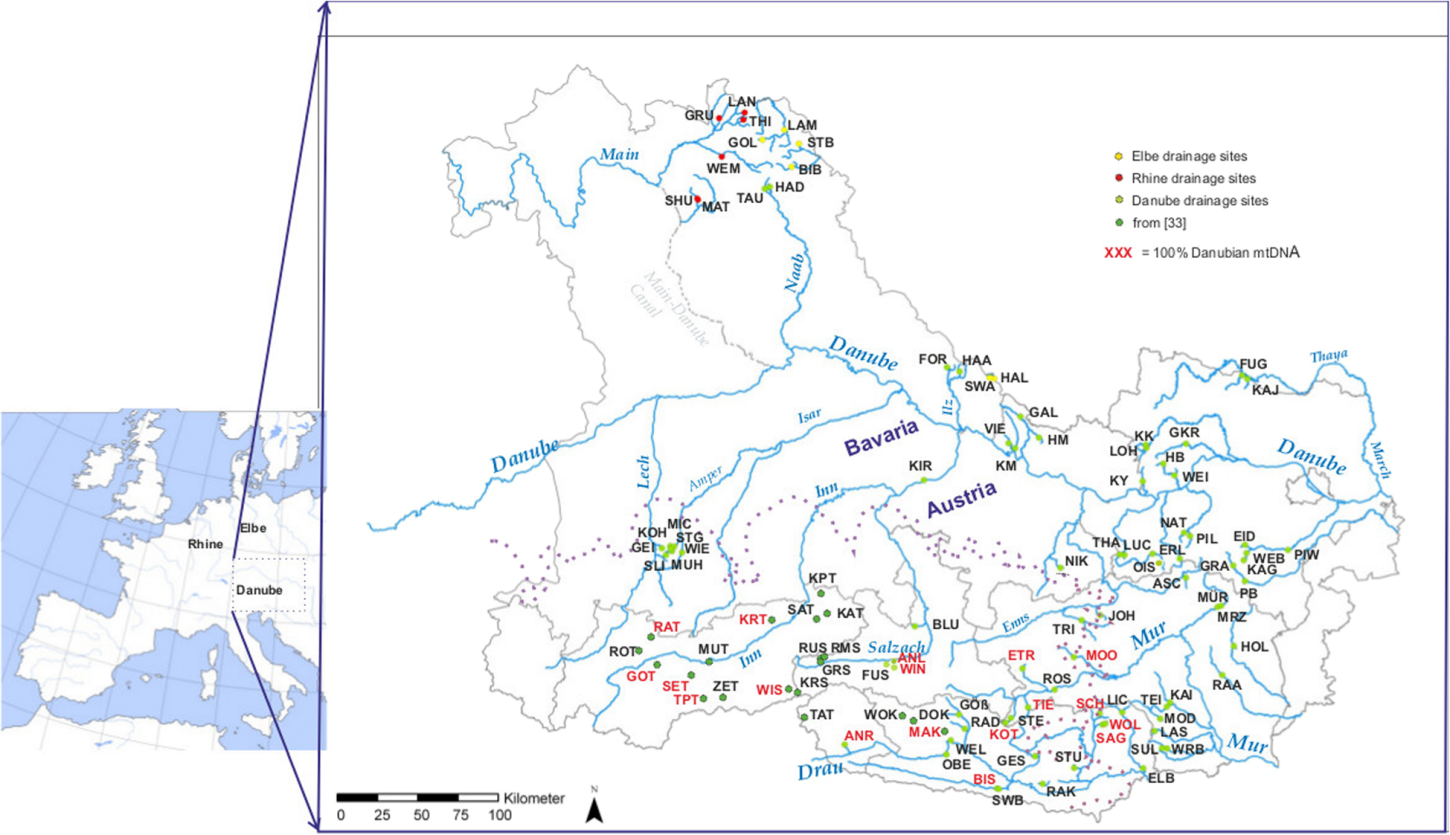
**Map of sample sites.** Map of all 97 sites sampled in this study as well as the 17 populations sampled in [30] and their corresponding drainages. The purple dotted lines correspond to the maximum extension of glacial ice at the height of the Würm glaciation [31]. Current national borders are shown with a light gray line; part of the border between Austria and the German State of Bavaria is defined by the Salzach and Inn rivers.

In this paper we characterize the population genetic structure in the upper Danube in the Alpine and sub-Alpine regions of Austria and Bavaria including comparison to samples from adjacent areas of the Rhine and Elbe drainages (Figure 1). This study was broadly designed to: 1) evaluate the hypothesis of [25] and [29] suggesting that the upper Danube in Austria and Germany may have been naturally colonized by the Atlantic lineage; 2) identify pure Danubian lineage populations for conservation purposes, and 3) to question the utility of river basin-based phylogeographic expectations versus biological hypotheses for explaining the extant distribution of genetic variation across large-scale aquatic landscapes. More specific hypotheses concerning the potential hybrid origins of groups of populations are presented below.

## Results

### MtDNA and LDH-C1 analysis

**Figure 2:**
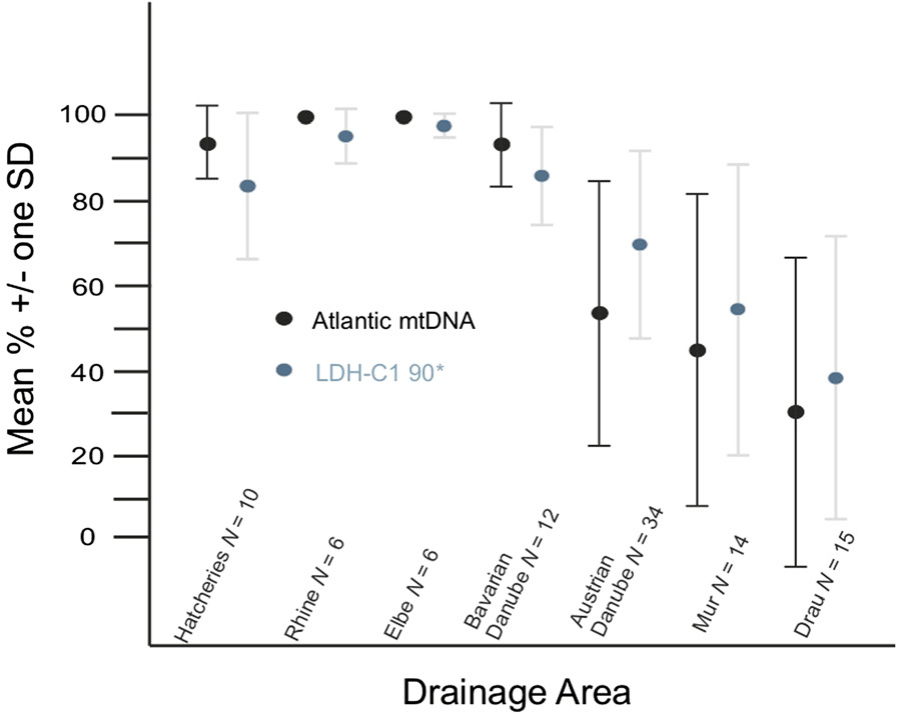
**Percentage of Atlantic basin specific markers (mtDNA** &LDH-C1 90*) in the 97 populations sampled in this study. The mean (plus S.D) percentage of Atlantic mtDNA and the LDH-C1 90* allele across the major drainages included all hatcheries as a group. *N* is the number of populations within each drainage.

Atlantic lineage mtDNA was fixed in the Rhine and Elbe drainages and there was a general decreasing trend in its mean occurrence from west to east (i.e. downstream) within the Danube basin ranging from a high of 93.5% in the Bavarian Danube to a low of 32.1% in the Austrian Drau, south of the Alps (Table 1; Figure 2). Although the percentage of Atlantic mtDNA varied widely among sample sites within the Danube catchment, in our data only previously glaciated regions within the Austrian Mur and Drau drainages revealed some populations (*N* = 11) fixed for Danubian mtDNA (Table 1). An analogous west–east trend in decreasing mean frequency was seen for the Atlantic basin LDH-C1 90* allele (Table 1; Figure 1), with near fixation in the Elbe (97.8%) and Rhine (95.5%) basins and the lowest occurrence in the Austrian Drau (37.3%). The alternate LDH-100* allele was fixed in five (ETR, MOO, BIS, SAG, WOL) of the 11 populations (ETR, KOT, MOO, TIE, ANR, BIS, SAG, SCH, WOL, ANL and WIS) that were fixed for the Danubian mtDNA lineage, but nowhere else (Table 1). Fixation of the LDH-C1 90* allele was found in only two populations in the Danube catchment (in Bavaria), and both were fixed for Atlantic mtDNA (Ilz & Lech, Table 1). There was a highly significant correlation (Kendall’s tau 0.609; *P* < 0.001) between the frequencies of Atlantic mtDNA and the LDH-C1 90* allele across all populations. Correcting for multiple testing, there was no deviation from HWE at the population level for the LDH-C1 locus. For some comparisons in the text we will refer to populations fixed for Danube mtDNA as “pure Danubian”.

**Table 1:** **Complete list of all 114 populations mentioned in this paper; 97 of these were newly analyzed for this study and 17 stem from**[30]

				**(WGS84)**			**(%)**				
**Population**	**Pop-Code**	**Drainage**	**Region**	**Lat.**	**Long.**	***N***	**mtDNA At**	LDH-90*	**Mgt.**	**MASL**	**Glac.**
Grumpelbach	GRU	**Rhine**/Main/Rodach/Kremnitz	Bav	50° 18′	11° 22′	19	100.00	100.00	3	393	No
Langenaubach	LAN	**Rhine**/Main/Rodach	Bav	50° 20′	11° 35′	21	100.00	100.00	1	630	No
Mathelbach	MAT	**Rhine**/Main/Wiesent/Leinleiter	Bav	49° 50′	11° 12′	32	100.00	93.94	2	456	No
Schulmühlbach	SHU	**Rhine**/Main/Wiesent/Leinleiter	Bav	49° 50′	11° 11′	27	100.00	83.33	1	333	No
Thiemitz	THI	**Rhine**/Main/Rodach/Wilde Rodach/Thiemitz	Bav	50° 17′	11° 35′	22	100.00	100.00	1	612	No
Weisser	WEM	**Rhine**/Main	Bav	50° 03′	11° 39′	24	100.00	95.65	3	485	No
**Sub-total**		**Rhine**	**Bav**			**145**	**100.00**	**95.49**			
Bibersbach	BIB	**Elbe**/Eger	Bav	50° 03′	12° 00′	22	100.00	100.00	3	640	No
Goldbach	GOL	**Elbe**/Saale/Sächsische Saale/Pulschnitz	Bav	50° 11′	11° 44′	23	100.00	95.83	1	584	No
Harlandtbach	HAL	**Elbe**/Moldau/Kalte Moldau	Bav	48° 51′	13° 44′	19	100.00	100.00	2	957	No
Lamitz	LAM	**Elbe**/Saale/Sächsische Saale	Bav	50° 14′	11° 56′	23	100.00	93.18	3	493	No
Steinselb	STB	**Elbe**/Eger	Bav	50° 10′	12° 03′	21	100.00	97.73	1	1040	No
Schwarzbach	SWA	**Elbe**/Moldau/Kalte Moldau	Bav	48° 51′	13° 42′	23	100.00	100.00	2	588	No
**Sub-total**		**Elbe**	**Bav**			**131**	**100.00**	**97.79**			
Forellenbach	FOR	**Danube**/Ilz/Große Ohe	Bav	48° 55′	13° 21′	21	100.00	100.00	1	754	No
Geiselmoosbach	GEI	**Danube**/Lech/Rottbach	Bav	47° 53′	10°58′	24	100.00	65.22	2	699	Yes
Haarauer Saige	HAA	**Danube**/Ilz/Kleine Ohe/Sagwasser	Bav	48° 54′	13° 28′	26	76.92	100.00	1	783	No
Haidenaab	HAD	**Danube**/Naab	Bav	49° 55′	11° 49′	26	85.00	90.74	2	507	No
Kirnbach	KIR	**Danube**/Inn	Bav	48° 17′	13° 9′	20	100.00	87.50	2	330	No
Kohlgraben	KOH	**Danube**/Isar/Amper/Ammersee/Rott	Bav	47° 54′	11° 02′	22	73.91	86.84	3	580	Yes
Milchelbach	MIC	**Danube**/Isar/Amper/Ammersee/Rott	Bav	47° 54′	11° 05′	22	86.36	85.00	3	568	Yes
Muhlbach	MUH	**Danube**/Isar/Amper/Ammersee/Rott	Bav	47° 52′	11° 03′	24	100.00	77.27	2	617	Yes
Schlittbach	SLI	**Danube**/Isar/Amper/Ammersee/Rott	Bav	47° 51′	11° 01′	23	100.00	65.91	2	676	Yes
Steingraben	STG	**Danube**/Isar/Amper/Ammersee/Rott	Bav	47° 53′	11° 03′	23	100.00	88.10	2	616	Yes
Tauritzbach	TAU	**Danube**/Naab	Bav	49° 54′	11° 46′	27	100.00	85.19	2	541	No
Wielenbach	WIE	**Danube**/Lech	Bav	47° 50′	10° 59′	22	100.00	100.00	2	755	No
**Sub-total**		**Bavarian Danube**	**Bav**			**280**	**93.52**	**85.98**			
Aschbach	ASC	**Danube**/Enns	Styr	47° 43′	15° 19′	34	76.92	83.33	2	900	No
Eidechselbach	EID	**Danube**/Fischa/Piesting	L_Aus	47° 54′	15° 49′	29	36.67	91.67	1	612	No
Erlauf	ERL	**Danube**	L_Aus	47° 50′	15° 17′	23	75.00	70.83	3	777	No
Fugnitz	FUG	**Danube**/March/Thaya	L_Aus	48° 51	15° 50′	28	100.00	98.21	3	446	No
Galgenbach	GAL	**Danube**/Große Mühle	U_Aus	48° 38′	13° 58′	19	13.33	97.37	3	538	No
Grosse Krems	GKR	**Danube**/Krems	L_Aus	48° 28′	15° 21′	39	79.49	93.59	3	654	No
Höllbach	HB	**Danube**/Weiten	L_Aus	48° 22′	15° 9′	27	33.33	62.96	2	900	No
Hollerbach	HOL	**Danube**/Raab	Styr	47° 20′	15° 42′	34	77.42	82.81	2	560	No
Hummelmühlbach	HM	**Danube**/Große Mühle/Steinerne Mühl	U_Aus	48° 31′	14° 7′	16	6.25	84.38	2	700	No
Johnsbach	JOH	**Danube**/Enns	Styr	47° 31′	14° 36′	33	72.00	74.00	2	1100	Yes
KaYesbach	KAJ	**Danube**/March/Thaya	L_Aus	48° 49′	15° 53′	25	96.43	91.07	2	443	No
Kaltergang	KAG	**Danube**/Fischa/Piesting	L_Aus	47° 49′	15° 48′	30	86.67	83.33	1	734	No
Kleine Mühl	KM	**Danube**	U_Aus	48° 28′	13° 55′	15	46.67	76.67	2	530	No
Kleine Ysper	KY	**Danube**/Ysper	L_Aus	48° 16′	14° 59′	20	40.00	87.50	3	475	No
Kleiner Kamp	KK	**Danube**/Kamp/Großer Kamp	L_Aus	48° 27′	15° 1′	96	48.45	77.84	2	750	No
Lohnbach	LOH	**Danube**/Kamp/Großer Kamp/Kleiner Kamp	L_Aus	48° 28′	15° 1′	33	16.67	68.18	1	776	No
Luckenbach	LUC	**Danube**/Ybbs	L_Aus	47° 51′	14° 49′	10	80.00	40.00	2	849	No
Natters (2004)	NAT	**Danube**/Pielach	L_Aus	47° 58′	15° 19′	40	52.50	81.25	3	594	No
Niklbach	NIK	**Danube**/Enns/Steyr/Paltenbach	U_Aus	47° 47′	14° 17′	29	3.45	44.64	2	929	No
Ois (2004)	OIS	**Danube**	L_Aus	47° 52′	15° 3′	59	52.54	62.50	3	621	No
Pielach	PIL	**Danube**	L_Aus	47° 57′	15° 22′	33	51.52	75.76	3	508	No
Piesting at Wöll.	PIW	**Danube/**Fischa	L_Aus	47° 49′	15° 48′	24	83.33	87.50	3	762	No
Preinerbach	PB	**Danube**/Leitha/Schwarza	L_Aus	47° 42′	15° 49′	29	51.72	84.48	3	499	No
Raab	RAA	**Danube**	Styr	47° 10′	15° 36′	28	76.67	79.31	3	419	No
Schwarza	GRA	**Danube**/Leitha	L_Aus	47° 48′	15° 42′	25	100.00	75.00	3	600	No
Thannergraben	THA	**Danube**/Ybbs	L_Aus	47° 52′	14° 47′	30	100.00	46.67	2	522	No
Triebenbach	TRI	**Danube**/Enns	Styr	47° 30′	14° 27′	34	47.22	45.71	2	700	No
Viehbach	VIE	**Danube**/Kleine Mühl/Daylesbach	U_Aus	48° 29′	13° 51′	20	73.68	67.50	2	700	No
Weißenbach	WEB	**Danube**/Fischa/Piesting	L_Aus	47° 51′	15° 50′	26	52.17	73.21	1	546	No
Weiten	WIE	**Danube**	L_Aus	48°′18′	15°′15′	40	52.50	78.75	2	372	No
**Sub-total**		**Austrian Danube**				**928**	**76.01**	**73.94**			
Etrachbach	ETR	**Danube**/Drau/Mur	Styr	47°′14′	13°′57′	30	0.00	0.00	1	1400	Yes
Kainach	KAI	**Danube**/Drau/Mur	Styr	47′2′	15°′10′	27	73.08	66.67	3	362	No
Kotalmbach	KOT	**Danube**/Drau/Mur/Turrach	Styr	46°′56′	13°′49′	27	0.00	11.29	1	1580	Yes
Lassnitz	LAS	**Danube**/Drau/Mur	Styr	46°′52′	15′2′	32	34.38	39.39	3	320	No
Modriachwinkelbach	MOD	**Danube**/Drau/Mur	Styr	46°′56′	15′5′	34	11.43	48.61	2	1000	No
Moosbach	MOO	**Danube**/Drau/Mur	Styr	47°′17′	14°′23′	35	0.00	0.00	1	1400	Yes
Mürz (Feistritz)	MÜR	**Danube**/Drau/Mur	Styr	47°′33′	15°′35′	30	80.00	80.00	3	615	No
Mürz	MRZ	**Danube**/Drau/Mur	Styr	47°′34′	15°′36′	29	82.76	86.67	3	637	No
Rosenbach	ROS	**Danube**/Drau/Mur	Styr	47′7′	14°′13′	30	100.00	98.15	2	781	Yes
Schwarze Sulm	SUL	**Danube**/Drau/Mur	Styr	46°′46′	15′8′	37	62.16	77.03	3	870	No
Steinbach	STE	**Danube**/Drau/Mur/Turrach	Styr	46°′57′	13°′51′	26	23.33	53.33	2	651	Yes
Teigitsch	TEI	**Danube**/Drau/Mur	Styr	47′0′	15′8′	39	65.00	93.42	3	566	No
Tiefbach	TIE	**Danube**/Drau/Mur	Styr	47′1′	14′2′	25	0.00	17.24	1	1400	Yes
Wiesenriegelbach	WRB	**Danube**/Drau/Mur	Styr	46°′46′	15′6′	20	81.25	81.25	2	969	No
**Sub-total**		**Austrian Mur**				**421**	**43.81**	**53.79**			
Anrasersee	ANR	**Danube**/Drau/Mühlbach	Tyrol	46°′48′	12°′31′	19	0.00	23.68	1	2523	Yes
Bach in der Schütt	BIS	**Danube**/Drau/Gail	Car	46°′34′	13°′45′	18	0.00	0.00	1	520	Yes
Elbach	ELB	**Danube**/Drau/Lavant	Car	46°′40′	14°′57′	23	100.00	95.45	2	780	No
Gesgerbach	GES	**Danube**/Drau/Tiebel	Car	46°′44′	14′4′	20	45.00	32.50	2	660	Yes
Gößbach	GÖß	**Danube**/Drau/Lieser/Malta	Car	46°′58′	13°′26′	20	20.00	70.00	2	980	Yes
Lichtengrabenbach	LIC	**Danube**/Drau/Lavant	Car	46°′59′	14°′46′	24	28.00	24.00	2	900	No
***Maisbach***	MAK	**Danube**/Drau/Radlbach/Lieser	Car	46° 55′	13° 27′	26	0.00	*NA*	1	1200	Yes
Oberallacher Bach	OBE	**Danube**/Drau	Car	46° 45′	13° 20′	16	100.00	84.21	3	786	Yes
Radlbach	RAD	**Danube**/Drau/Lieser	Car	46° 54′	13° 29′	20	20.00	25.00	2	1080	Yes
Rakoutzabach	RAK	**Danube**/Drau/Gurk/Glan	Car	46° 35′	14° 7′	9	90.00	90.00	3	550	Yes
Saggrabenbach	SAG	**Danube**/Drau/Gurk/Görtschitz	Car	46° 55′	14° 37′	10	0.00	0.00	1	1080	Yes
Schafgrabenbach	SCH	**Danube**/Drau/Gurk/Görtschitz	Car	46° 58′	14° 35′	20	0.00	5.00	1	1066	No
Stieger Wiesenbach	SWB	**Danube**/Drau/Gail	Car	46° 34′	13° 46′	16	5.56	11.11	2	520	Yes
Stutterner Bach	STU	**Danube**/Drau/Gurk	Car	46° 40′	14° 23′	13	7.69	58.33	1	480	Yes
***Trojer Almbach***	TAT	**Danube**/Drau	Tyrol	46° 57′	12° 17′	15	100.00	NA	1*	1989	Yes
Wellenbach	WEL	**Danube**/Drau	Car	46° 50′	13° 24′	15	20.00	40.00	2	554	Yes
***Woisgenbach***	WOK	**Danube**/Drau	Car	47° 00′	13° 08′	30	43.00	NA	2	1300	Yes
Wolfsgrabenbach	WOL	**Danube**/Drau/Gurk/Görtschitz	Car	46° 55′	14° 38′	17	0.00	0.00	1	1140	No
**Sub-total**		**Austrian Drau**				**331**	**32.10**	**37.28**			
***Kapellenbach***	KPT	**Danube/Inn**	Tyrol	47° 39′	12° 23′	20	85.00	*NA*	2	594	Yes
***Katzbach***	KAT	**Danube/Inn**	Tyrol	47° 33′	12° 25′	20	15.00	*NA*	1	706	Yes
***Kreuzbach***	KRT	**Danube/Inn**	Tyrol	47° 28′	11° 51′	16	0.00	*NA*	1	561	Yes
***Mühlauer Bach***	MUT	**Danube/Inn**	Tyrol	47° 17′	11° 25′	12	100.00	*NA*	2	707	Yes
***Rappenbach***	RAT	**Danube/Inn**	Tyrol	47° 23′	10° 56′	20	0.00	*NA*	1	1169	Yes
***Rossbach***	ROT	**Danube/Inn**	Tyrol	47° 18′	10° 52′	30	77.0	*NA*	2	967	Yes
***Sandtalbraben***	SAT	**Danube/Inn**	Tyrol	47° 32′	12° 18′	20	25.00	*NA*	2	950	Yes
***Sendersbach***	SET	**Danube/Inn**	Tyrol	47° 10′	11° 15′	31	0.00	*NA*	1	1726	Yes
***Trins-Padast***	TPT	**Danube/Inn**	Tyrol	47° 04′	11° 24′	20	0.00	*NA*	1	1248	Yes
***Zeischbach***	ZET	**Danube/Inn**	Tyrol	47° 02′	11° 34′	17	12.00	*NA*	2	1339	Yes
Anlaufbach	ANL	**Danube/Inn/Salzach**	Szbg	47° 10′	13° 54′	65	0.00	19.00	2	1384	Yes
***Grubingerbach***	GRS	**Danube/Inn/Salzach**	Szbg	47° 17′	12° 24′	17	53.00	*NA*	1	925	Yes
***Krimmler Ache***	KRS	**Danube/Inn/Salzach**	Szbg	47° 05′	12° 13′	18	5.00	*NA*	2	1580	Yes
***Rettenbach_1***	RMS	**Danube/Inn/Salzach**	Szbg	47° 07′	12° 26′	20	50.00	*NA*	1	1158	Yes
***Rettenbach_2***	RUS	**Danube/Inn/Salzach**	Szbg	47° 18′	12° 25′	18	100.00	*NA*	1	1300	Yes
Winbach	WIS	**Danube/Inn/Salzach**	Szbg	47° 07′	12° 11′	31	0.00	34.00	2	1882	Yes
Blühnbach	BLU	**Danube/Inn/Salzach**	Szbg	47° 28′	13° 5′	40	10.00	26.25	2	911	Yes
Fuscher Ache	FUS	**Danube/Inn/Salzach**	Szbg	47° 09′	12° 48′	37	3.00	14.00	1	222	Yes
**Sub-total**		**Austrian Inn**				**452**	**29.7**	**23.31**			
A		**Hatchery**				29	85.19	82.76			
B		**Hatchery**				30	74.07	51.67			
C		**Hatchery**				24	100.00	100.00			
D		**Hatchery**				30	100.00	100.00			
E		**Hatchery**				31	100.00	77.42			
F		**Hatchery**				40	100.00	98.75			
G		**Hatchery**				30	96.00	90.00			
H		**Hatchery**				26	95.83	74.00			
I		**Hatchery**				27	100.00	100.00			
J		**Hatchery**				29	89.29	60.34			
**Sub-total**		**Hatcheries**				**296**	**94.04**	**83.49**			
**Grand total**		**All sample groups**				**2984**	**67.74**	**71.79**			

Populations from [30] are shown in bold italics. Listed is the drainage hierarchy, geo-political region (*Bav* Bavaria, *Styr* Styria, *L_Aus* Lower Austria, *U_Aus* Upper Austria, *Car* Carinthia, *Szbg* Salzburg), geographic coordinates (*Lat.* Latitude, *Long.* Longitude), *N* = number of individuals, *(%) mtDNA* percentage of Atlantic mtDNA,% LDH-90* percentage of the LDH-90* allele, *Mgt* management level (*1* unstocked, *2* possible introductions or immigration from managed reaches, *3* definitely stocked), *MASL* meters above sea level, *Glac* previously glaciated (yes, no). Pure Danubian populations in Carinthia with low samples sizes (BIS, SAG, SCH, & WOL) have all been recently validated with increased sample sizes (Weiss, unpublished data).

### Microsatellites

The mean allelic richness across all loci at the population level ranged from 2.5 (BIS, Drau drainage) to 7.9 (SUL, Mur drainage, Additional file 1). This richness was significantly different (Kruskal-Wallis test; *P* = 0.018) among drainages with the highest values found in the Austrian Danube (6.4) and the lowest in the Austrian Drau (5.1). There was a highly significant difference (Mann–Whitney U, *P* < 0.001) in mean allelic richness between populations fixed for Danubian mtDNA (3.8) and the remaining Danubian populations of the Mur and Drau drainages (6.2). *H*_E_ likewise ranged widely across populations (0.374 to 0.836) and showed wholly analogous patterns of statistical significance within and among major basins (data not shown). There was a significant positive correlation between *H*_E_ and the percent occurrence of Atlantic basin mtDNA across populations in the Austrian Mur (R^2^ = 0.488, *P* < 0.01) and the Austrian Drau (R^2^ = 0.391, *P* < 0.05) drainages supporting the influence of recent admixture; in contrast, no such correlation was found across populations in the Bavarian or the Austrian Danube.

Most populations (*N* = 74, 80%) showed no deviation from HWE, not considering table-wide corrections for multiple testing. The remaining samples that showed significantly negative *F*_IS_-values stem either from hatcheries or small headwater streams. Samples that showed significantly positive *F*_IS_-values are known to be either moderately or heavily managed. After correcting for multiple testing only a single heavily managed population (Schwarza, lower Austria) deviated significantly from HWE, with a positive *F*_IS_-value, presumably reflecting the presence of both wild and hatchery-origin fish.

The global *F*_ST_ across all 97 populations was 0.116 indicating a moderate level of differentiation among populations. Among drainage (Elbe, Rhine, Bavarian Danube, Austrian Danube, Mur and Drau) differentiation was considerably lower (*F*_ST_ = 0.031) while within drainage differentiation varied widely with the lowest values revealed among populations within the Bavarian Rhine (*F*_ST_ = 0.044) and Elbe (*F*_ST_ = 0.068), and the highest values found among populations within the Mur (*F*_ST_ =0.1465) and Drau (*F*_ST_ =0.188) where pure Danubian populations were found. A maximum *F*_ST_ value of 0.228 was observed among pure Danubian populations, presumably reflecting long periods of isolation relative to other among population comparisons within the global data set.

Using analog *F*-statistics (Φ), the AMOVA drainage model (Rhine, Elbe, Bavarian Danube, Austrian Danube, Mur and Drau) revealed 2.6% of the genetic variance distributed among drainages (Φ_CT_), 10% among populations within drainages (Φ_SC_), and 87.4% within populations (Φ_ST_), all of which were highly significant (P < 0.0001). The portion of among drainage variance (Φ_CT_) rose to 4.8% when using *R*-statistics reflecting the influence of mutation on the among drainage variance. A higher (Φ_CT_) value (13.8%) was seen based on a reduced model for differentiation between native Danube and remaining Danubian populations reflecting the relatively high divergence of pure Danubian populations within the global data set.

**Figure 3:**
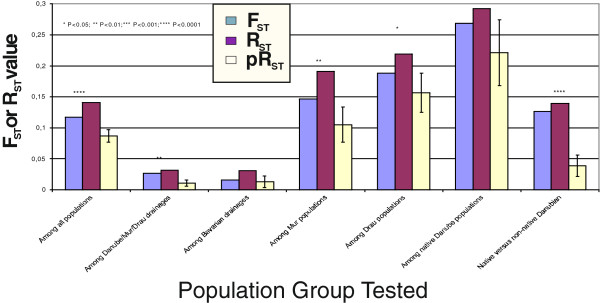
**Global comparisons of *F***_**ST **_**and *R***_**ST**_**.** Global comparisons of *F*_ST_ and *R*_ST_ using the allele size permutation test carried out it SPAGeDi 1.2. Comparisons include among all populations, among Danube, Mur, and Drau drainages, among the three Bavarian drainages (Danube, Elbe, Rhine), among all Mur populations, among all Drau populations, among all pure Danubian lineage populations, and pure-Danubian versus all other Danubian populations.

The allele size permutation test further supported the influence of mutation on phylogeographic structure (and thus longer periods of isolation) for global comparisons (e.g. among all populations, or among all drainages) as well as those involving among population differentiation of the native Danube populations (Figure 3). In contrast, non-mutational mechanisms (drift and gene flow) were presumably dominant in comparisons involving Bavarian populations, supporting a surprising lack of geographic structure across the sampled sites of the three major drainages (Rhine, Elbe, Danube) in this region.

**Figure 4:**
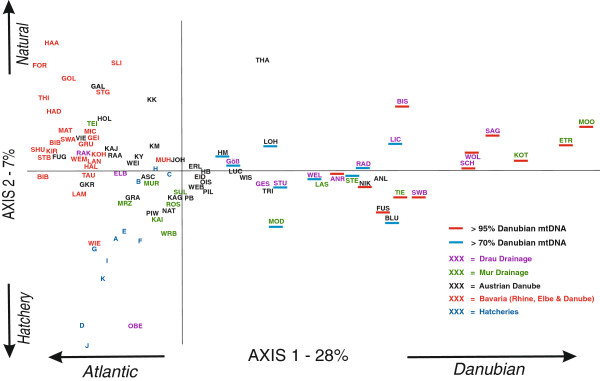
**PCA of microsatellite frequency data.** Principal components analysis based on microsatellite allele frequency data from 97 populations. Populations are colour-coded by major drainage, including hatcheries as a group. Populations carrying > 95% (blue bar) or between 70% and 95% (red bar) Danubian lineage mtDNA are additionally marked.

The principal component analysis of populations based on microsatellites revealed one axis (PC-1, 28.2%) generally reflecting population differentiation corresponding to the two mtDNA (Atlantic and Danubian) lineages (Figure 4). Populations fixed for, or revealing high percentages of Danubian mtDNA occur to the right along the x axis, whereas those with very low frequencies are to the left. The second axis (PC-2, 7.2%) tends to distinguish among populations within the Atlantic mtDNA lineage; hatchery populations are at the low end of the axis, whereby Bavarian populations (red text) are at the high end and Austrian Danube populations (black text) are generally intermediate (Figure 4). The location of the OBE (Oberallacher Bach) population from the Drau drainage at the low end of this axis is concordant with fixation of Atlantic lineage mtDNA despite its south Alpine location. Subsequent to our genetic screening of this population, local authorities told us that this river stretch was fishless (above a waterfall) and was stocked by an unknown source. Our data imply that the source of stocking was a pure Atlantic hatchery strain. All additional PCA factors reflected only minor portions of variance in the data set, and were neither biologically or geographically interpretable at the global scale (Additional file 2).

Two of the ten hatcheries (B &C) have an intermediate position. The first is known to be supplemented with locally caught fish, while the second is of Czech origin and presumed to be founded with fish from the Elbe drainage. A few Austrian Danube populations, all north of the Danube (e.g. KK, THA, GAL, VIE), are located intermediate along the X-axis, suggesting some admixture with pure Danube-lineage fish. A large number of Austrian Danube populations are found around the center of both axes potentially reflecting admixture between one or more lineages.

Structure analysis revealed three main clusters primarily corresponding to: (1) Bavarian populations irrespective of drainage; (2) the so-called pure Danubian populations from the Mur and Drau; and (3) remaining populations primarily from the Danube catchment (data not shown). However, only the pure Danubian populations showed a high mean proportion of self-assignment (based on mean q-values) (Table 2) (Additional file 3). Within other geographic groups, the population-level q-values (data not shown) varied widely, and some individual populations were assigned to groups outside of their region. For example, several Austrian populations north of the Danube could be assigned to the Bavarian group (e.g. the mean Q value for VIE = 0.707, HM = 0.699, KK = 0.779, and HOL = 0.702). Likewise, a number of Bavarian populations, including one from the Elbe (LAM) could be assigned to the third cluster representing hatcheries. While K = 3 showed the highest delta K, K = 4 also revealed a biologically meaningful group consisting of five Austrian populations (KK, HM, KM, GAL & VIE) all from the granitic regions (Waldviertal & Muhlviertal) north of the Danube, thus providing some level of distinction from Bavarian populations.

**Table 2:** Average proportion of membership (q-values) of wild populations within major drainages to the different clusters defined by STRUCTURE using K = 3

**Drainage**	**Cluster 1**	**Cluster 2**	**Cluster 3**
	**Bavarian group**	**Pure-Danubian group**	**Hatchery group**
Elbe	**0.63 (0.22)**	0.04 (0.03)	0.33 (0.22)
Rhine	**0.73 (0.16)**	0.04 (0.01)	0.24 (0.15)
Bavarian Danube	**0.62 (0.23)**	0.05 (0.02)	0.33 (0.24)
Austrian Danube	0.37 (0.18)	0.18 (0.14)	**0.45 (0.16)**
Mur	0.23 (0.14)	0.24 (0.18)	**0.53 (0.15)**
Drau	0.21 (0.13)	**0.39 (0.28)**	**0.40 (0.23)**
Pure-Danubian	0.06 (0.04)	**0.88 (0.11)**	0.06 (0.07)

Standard deviations are shown in parentheses and the highest value for each drainage is shown in bold.

**Figure 5:**
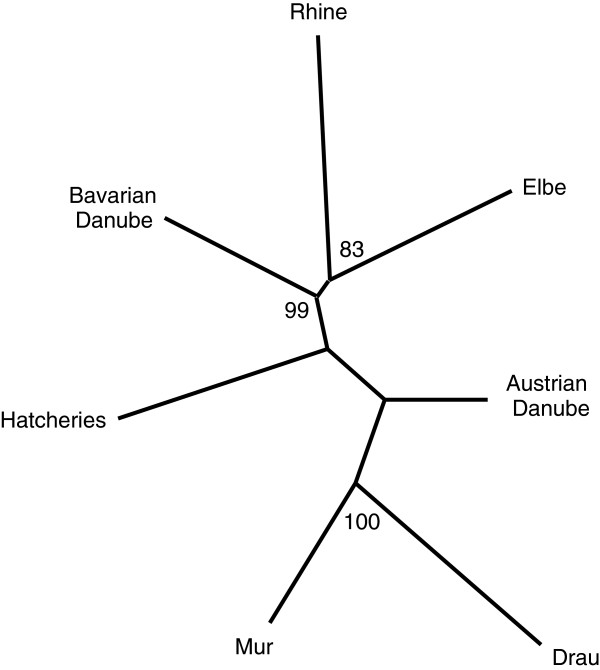
**NJ – tree of populations grouped by drainage.** Neighbour-Joining tree of populations (grouped by drainage) based on Dc chord distances of microsatellite allele frequency data. Support values were based on bootstrapping over loci (500 replicates).

Statistically significant but weak isolation-by-distance signals were seen for Bavaria (R^2^ = 0.029, *P* = 0.004), the Austrian Danube (R^2^ = 0.075, *P* = 0.026) and pure Danubian populations (R^2^ = 0.438, *P* = 0.003) as well as the Drau drainage alone (Drau R^2^ = 0.172, *P* = 0.026), suggesting that natural as opposed to anthropogenic mechanisms are still operative, if not dominant, in determining among population differentiation. The population tree highly supported both the distinction of Bavaria relative to Danube populations (including hatcheries), and the two members of a south alpine group in the Danube drainages (Mur and Drau). In addition, it provided moderate support for a group containing the Rhine and Elbe catchments (Figure 5). A weak but statistically significant signal for demographic expansion was seen for the Rhine basin (*P* = 0.04), as well as the Rhine and Elbe basins combined (*P* = 0.04) using the within-locus k test. This test is noted for being more sensitive to recent as opposed to ancient expansion events compared to the inter-locus g test, which did not reveal any statistically significant signal.

### Global patterns of admixture

**Figure 6:**
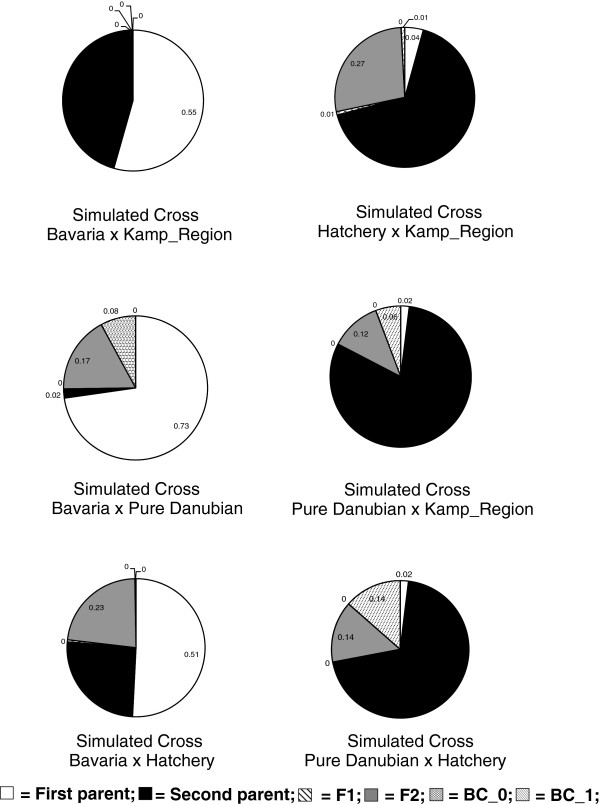
**Assignment of Austrian Danube individuals based on simulated crosses.** Mean posterior probability that individual samples from the Austrian Danube belong to the parental or hybrid genotype categories listed, for each of six simulated crosses. BC_0 is a first generation backcross to the first parent of the simulated cross, and BC_1 is a first generation backcross to the second parent of the simulated cross. The Kamp_Region represents populations in crystalline river sites in Northeast Austria (i.e. KK, HM, KM, GAL and VIE).

**Figure 7:**
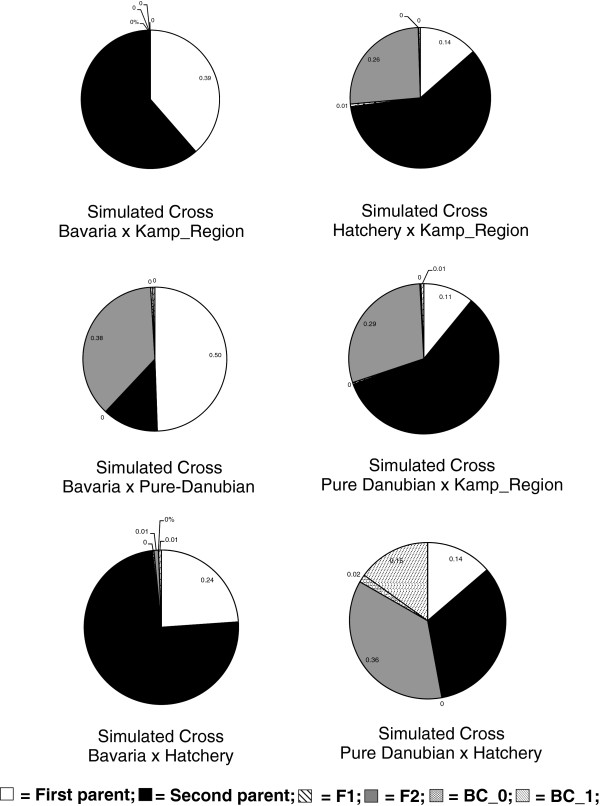
**Assignment of Mur and Drau individuals based on simulated crosses.** Mean posterior probability that individual samples from the Mur and Drau drainages belong to the parental or hybrid genotype categories listed, for each of six simulated crosses. BC_0 is a first generation backcross to the first parent of the simulated cross, and BC_1 is a first generation backcross to the second parent of the simulated cross. The Kamp_Region represents populations in crystalline river sites in Northeast Austria (i.e. KK, HM, KM, GAL and VIE).

To generate hypothetical sources of admixture, we simulated six different crosses between four population groups (identified with Structure): 1) Hatcheries, 2) pure Danubian, 3) Bavarian, 4) Northeast Austrian granitic group (KK, HM, KM, GAL & VIE). There were three presumably admixed population groups of interest: 1) the Austrian Danube, 2) the Mur, and 3) the Drau. For each of these three groups, we applied Bayesian clustering tests to each of the simulated hybrid crosses, resulting in 18 tests. Across all 18 tests, the posterior probably of individual assignment to an F1 hybrid was negligible (<1%) (Figures 6 &7). However, meaningful levels of individual assignment to subsequent classes (i.e. F2 and backcrosses) were seen for the Austrian Danube group (Figure 6) as well as the Mur and Drau (Figure 7). The percentage assignment to a post-F1 class for Austrian Danube individuals reached 28 and 29% for the two simulated crosses involving hatcheries and other Austrian population groups (i.e. native Danube & granitic North). For the Mur and Drau individuals, assignment to a post-F1 class reached a high of 53% for the simulated cross between hatcheries and the pure Danubian populations (Figure 7).

## Discussion

Multiple marker systems (mtDNA, microsatellites, LDH-C1) strongly support the natural occurrence of the Atlantic lineage of brown trout throughout large areas of the upper Danube catchment, whereas there is no evidence of the Danube lineage in the sampled populations of the Bavarian Rhine and Elbe catchments and very little trace (< 7% mtDNA) of the Danube-lineage in the sampled portion of the Bavarian Danube. The cause of this large-scale pattern is natural, reflecting the admixed faunal character of the upper Danube established over both geological and glacial time scales [9]. These results are largely concordant with the distribution of the Atlantic lineage of brown trout in Czech and Slovakian tributaries of the upper Danube [32], but these authors did not argue for natural colonization. While the timing and colonization corridors of especially north-to-south colonizing fish taxa (i.e. from Atlantic to the Danube basin) are poorly understood, it is clear that the genera *Salvelinus* and *Coregonus*[33] have arrived in the upper Danube relatively recently during the last (Würm) or penultimate (Riss) ice age. As divergence for brown trout across the Rhine/Danube/Elbe contact zones are minimal to non-existent, we presume the species arrived in the upper Danube from the north during these ice age transitions. However, we emphasize that described river capture events are primarily in the opposite direction (i.e. from the Danube into the Atlantic basin), and this corresponds well with the paleo-hydrological perspective that the Danube basin has been continuously loosing area to the Rhine and Elbe basins across the ice ages [34].

While a relatively clear gradient of increasing Danube-specific alleles is found with increasing distance downstream (west-to-east) in the Danube catchment, the source of admixture displays a geographic as well as glacial pattern. Hatchery Atlantic strains appear to be the source of admixture in glaciated regions, especially south and southeast of the Alps, whereas natural sources of Atlantic lineage colonization from the upper Bavarian Danube appear to be the primary source of admixture north and northeast of the Alps, especially in unglaciated regions. Nonetheless, the influence of stocking in the region can be evaluated at a population level, as hatchery and wild Atlantic genotypes can be distinguished, but the overall presence and dominance of Atlantic genotypes is based on natural colonization.

Regardless of the orientation of the Alps, pure Danube lineage populations are exceedingly rare and appear limited to isolated high elevation populations in previously glaciated areas. This inference is further supported by an earlier study reporting 72% Danube lineage mtDNA and nine populations (three reported herein) fixed for Danube-lineage mtDNA in the upper Inn, Lech, Salzach and Drau drainages in Austria [30]. Curiously, all occur within previously glaciated areas and all over 1000 meters above sea level (MASL) (Table 1; Figure 1). The distribution of populations with 100% Danube-linage mtDNA form a peri-alpine ring that is difficult to explain based on either purely anthropogenic influence or any simple natural mechanism of colonization. Since the analysis of this data set, three additional pure Danubian-lineage populations have been identified, all on the south slopes of the Alps in Carinthia, and all over 1000 MASL (Weiss, unpublished data).

Brown trout undoubtedly occupied large areas of Austria even during the height of the Würm glaciation, as glaciers did not reach the Danube from the south. No real glaciation occurred either north of the Danube or across large areas east and southeast of the Alps. In the mountainous region of Salzburg, for example, glaciers reached a maximum extension of about 5–600 MASL approximately 24–22,000 years ago [35]. Based on accessibility, it is assumed that cold-tolerant fish re-colonized the Salzach River catchment post-glacially from the Danube and its tributaries to a maximum extent ca.11,000 year ago [35] during the early Holocene warm period. Between 11 and 4,000 years ago depositional and erosional processes created numerous impassible barriers isolating cold-tolerant fishes, namely brown trout and sculpin (*Cottus gobio*) in some river reaches found between 1 and 2,000 MASL today. We assume that this scenario was similar for many other glaciated river valleys of the Austrian Alps. Thus, pure Danubian lineage brown trout are primarily, if not exclusively found in headwater tributaries that were accessible in the early Holocene, but are now physically isolated from downstream colonization.

For regions south and southeast of the Alps, Atlantic lineage trout apparently exist exclusively through human-mediated mechanisms. That the admixture in these regions is more recent than to the north is strongly supported by the elevated levels of allelic diversity in the presence of both mtDNA strains in the Mur and Drau compared to the lack of such a correlation in the north. However, elsewhere the existence of pure Danubian lineage brown trout in isolated headwater systems means that they colonized these systems in the early stages of the current interglacial in the absence of the Atlantic lineage. This is difficult to explain as the Atlantic lineage not only dominates brown trout stocks in the upper Danube in Bavaria, but also reveals no signal of recent (i.e. post-glacial) expansion and thus must have been available for post-glacial colonization of the Alps. There is also no indication of a breeding barrier between Danubian and Atlantic lineages. Thus, the post-glacial expansion into alpine rivers of only one of these two co-existing lineages strongly implies some level of pre-glacial physical separation within the upper Danube. Interestingly, some authors have considered the Atlantic lineage brown trout stocks in the region as *Salmo trutta*, but the Danubian linage brown trout stocks in the upper Danube as *Salmo labrax* admitting a hybrid zone in the upper Danube [24]. Regardless of one’s taxonomic viewpoint, we postulate that the Danube lineage of brown trout must have existed in numerous small-scale peri-Alpine refugia and were the initial colonizers of at least some Alpine streams following glacier retreat. Such a scenario begs the question of whether the Danube lineage is more adapted to steeper gradient or glacially influenced habitats. Increasing attention is being paid to finer than basin-scale local adaption of brown trout [36]. Keller et al. [37] investigated the possibility of an elevation gradient being associated with outlier loci showing some signs of divergent selection within drainages but the association with elevation were weak, and the Danube lineage was essentially not involved in their work. The existing isolated populations of the pure Danubian lineage in high elevation habitats of Austria provide an intriguing source of material for future experiments on their potential for adaptive advantage compared to the Atlantic lineage. In contrast, one could pose the hypothesis of whether or not the Atlantic lineage has an adaptive advantage over the Danube lineage in the lower elevation river courses.

Regardless of such experimental avenues, it is apparent that a phylogeographic paradigm for European brown trout is failing to provide both researchers and managers with the proper framework for providing biological insights. From a purely management standpoint, the identification of a “Danubian” or an “Atlantic” genetic lineage is useless if not misleading in many (but not all) areas of the Danube basin, because the so-called Atlantic lineage is the dominant native trout lineage in much if not all of the Bavarian Danube and overlaps extensively in many drainages of Austria as well. Moreover, a homogeneous view of the Danubian lineage for management purposes might very well be counter-productive if strong selective mechanisms are operative in higher elevation populations. Indeed, elevation as a proxy for specific environmental conditions may turn out to be an important functional characteristic at the population or meta-population level for conservation purposes. Discarding or lumping admixed populations might also be counter-productive if some regions have arrived at this admixture through human-mediated events, while others have been admixed for thousands of years, representing unique and well-adapted gene pools. Our results and baseline data allow us to distinguish not only between native and anthropogenic introgression within the Atlantic lineage, but also the relict status of peri-alpine Danubian populations providing a valuable resource for future conservation planning.

The initial naming and description of five major mtDNA lineages in brown trout [13] was a breakthrough for European phylogeographic studies. Numerous subsequent studies have added detail and some controversy as to the origin of these lineages, but to date no study has provided a single biologically relevant character that is fixed or even predominant in one or more of these so-called lineages. Considering the high-level of physical fragmentation of all brown trout populations, it may be time to question the usefulness of the mtDNA phylogeographic lineages in management schemes, but also in the logical construction of basic science research programs. The universality of this concern related to other phylogeographically circumscribed lineages of plants and animals can be debated, but for highly fragmented species with large ranges it might indeed be broadly applicable, as recently suggested for European grayling [38], another pan-European salmonid species.

## Conclusions

The Atlantic lineage of brown trout is native to large areas of the upper Danube in Bavaria where it predominates, but also in numerous drainages of the Austrian Danube. The lineage has been a very successful post-glacial invader of river courses of the Austrian Danube, but to date there is no evidence of natural occurrence south and southeast of the Alps in the provinces of Styria and Carinthia. Pure Danubian lineage populations are found primarily in previously glaciated regions at higher elevations, in a peri-alpine distribution implying that the lineage colonized headwater river courses post-glacially ahead of the expansion of the Atlantic lineage. The distribution pattern of these two lineages is difficult if not impossible to describe without invoking strong biological mechanisms, meaning that a purely phylogeographic paradigm for these lineages fails to explain their distribution in the upper Danube basin. More recently, anthropogenic activities have aided the spread of the Atlantic lineage and there is no clear evidence of niche segregation or a breeding barrier where the two lineages are found in sympatry today.

## Methods

### Samples

A total of 2568 brown trout were sampled from 97 populations (including 10 hatcheries) across Austrian and Bavarian reaches of the upper Danube basin, as well as Bavarian reaches of the upper Elbe and Rhine catchments (Table 1; Figure 1). Fish were sampled with certified back-pack electric fishing generators, licensed operators, and with written permission from the local (district) level authorities as required by law. Small (1.2 mm) samples were cut from regenerative caudal fin tissue and stored in 96% ethanol. All fish were released unharmed back into their respective habitats. A wide variety of natural and relatively intact habitats were sampled including small headwaters with little or no known history of stocking as well as moderate to heavily managed fisheries. For post-hoc descriptive purposes, each population was given a three scale rating in relation to known management history: 1) no known history of stocking and unlikely affected by dispersal from managed waters; 2) at least one known event of stocking and/or lack of isolation from managed waters: 3) known history of stocking and management. Genomic DNA was extracted from fin clips using a high-salt extraction technique [39]. For some analyses and inferences concerning mtDNA only, published data [30] from an additional 17 populations primarily from the upper Inn drainage in western Austria were also used (Table 1; Figure 1). Thus the total number of populations integrated into the inferences of this study is 114, whereas results of 97 of these are reported for the first time.

### Genetic analysis

Microsatellite analysis was based on a two reaction, 12 locus multi-plex assay specifically developed and optimized for the region as previously reported [40]. SSR profiles were recorded using the GeneMapper Software v3.7 (Applied Biosystems). Following evaluation with the program Micro-Checker 2.2 [41], one locus (Ssa85) was removed from the analysis due to the unambiguous presence of null alleles. Variation at two additional markers (mtDNA and LDH-C) was evaluated due to their broad-scale phylogeographic information content. Raw allelic data for all loci and populations is provided [Additional file 4].

#### mtDNA

As both the Atlantic and Danubian mtDNA lineages of browns trout exist in the region [13,14,25], we developed an allele-specific PCR assay to assign an individual’s mtDNA to one of these two lineages. A multi-plex allele-specific assay was developed to screen two diagnostic positions in the control region (positions 26 & 389 in [27]). One primer (Da26 –GACTTTTCAGCTATGTACAATAACAAA) was combined with three published primers (LN19 and HN20 [13]; and 28ribaF [40]) to produce diagnostic bands for the Atlantic (452 bp) and Danubian (1035 bp) lineages. The PCR was performed in a total volume of 10 μl with 1X reaction buffer (PeqLab), 0.2 mM of each dNTP, 0.06 μM of primer At389Rd, 0.4 μM of primer Da26 + 2Fb, 0.15 μM of primer CytR, 0.03 μM of primer 28ribaF, 1.1 mM of MgCl_2_, 0.4 U Taq polymerase (PeqLab) and 20–50 ng genomic DNA. The reaction consisted of 3 min denaturation at 94°C, 32 cycles of 45 s at 94°C, 15 s at 61°C, 30 s at 72°C, and a final extension step of 7 min at 72°C. The reactions were loaded on a 2% agarose gel.

#### LDH-C1

We developed an allele-specific duplex assay to replace the PCR-RFLP assay from [42] in order to efficiently screen the bi-allelic phylogeographically informative LDH-C1 locus [43]. We selected two primers (Ldhxon4F-100 and Ldhxon4R-At) with the 3′end located on the allele-defining substitution, which in combination with Ldhxon3F and Ldhxon4R [42] allowed detection of all three relevant genotypes in one PCR. PCR was performed in a total volume of 10 μl with 1X reaction buffer (PeqLab), 0.2 mM of each dNTP, 0.8 μM of primer Ldxon4F-100 (ATTGTTCTCCCACGGTCAGA), 0.1 μM of primer Ldxon4R-At (GTTCGCCGTCACAGAGTAGC), 0.03 μM of primer Ldhxon3F, 0.03 μM of primer Ldhxon4R, 1.8 mM of MgCl_2_, 0.8 U Taq polymerase (PeqLab) and 20–50 ng genomic DNA. The reaction consisted of 3 min denaturation at 94°C, 32 cycles of 45 s at 94°C, 10 s at 70°C, 30 s at 72°C, and a final extension step of 7 min at 72°C. The reactions were loaded on a 3.8% NuSieve GTG agarose gel (Cambrex Bio Science). Amplified fragments of 340 bp and 100 bp characterize the *90 and *100 alleles, respectively, while an additional 440 bp fragment spanning regions of exons 3 and 4, as described in [42] is also observed.

### Data analysis

The number of alleles per locus, allelic richness, observed (*H*_O_) and expected (*H*_E_) heterozygosity and tests for deviations from Hardy-Weinberg expectations (HWE) were performed with FSTAT 2.9.3.2 [44]. The distribution of genetic variation across major drainage basins was evaluated with an hierarchical analysis of genetic variation (AMOVA) [45] using Arlequin v 3.11 [46] whereby a number of geographic partitions were evaluated using both *F*_ST_- and *R*_ST_-statistics. The contribution of microsatellite allele sizes (i.e. a measure reflecting mutation) to the distribution of this variation was examined using the allele size permutation test [47] implemented in SPAGeDi 1.2 [48]. The distribution of *R*_ST_ values from 10 000 permutations (p*R*_ST_) was compared to the observed *R*_ST_, which is analogous to *F*_ST_ when mutations are not contributing to genetic differentiation, and significantly greater than *F*_ST_ when they do. Such an evaluation should allow more temporal insight into the comparison of pairwise divergence estimates among groups of populations and whole basins. To gain perspective on the overall genetic relationships among the large number of populations screened we also carried out a Principal Component Analysis (PCA) on gene frequency data using the software PCAGEN 1.2 [49]. Populations were also grouped into drainages and unrooted Neighbor-Joining trees were constructed using the program Populations 1.2.30 [50]. Population trees were constructed based on the Dc chord distance [51]. Node confidence was evaluated by bootstrapping over loci (500 replicates).

An a priori evaluation of genetic structure across the global data set was carried out with the program Structure v2.1 [52] assuming admixture and independent allele frequencies. For each simulated K, the first 20,000 steps were discarded as burn-in, followed by 100,000 iterations to collect the data. We derived the most likely value of K using the second-order rate of change L”(K) following [53] using the on-line tool Structure Harvester (http://taylor0.biology.ucla.edu/struct_harvest). Structure analysis was repeated separately for a number of population groups defined by major drainages or sub-drainages (i.e. Rhine, Elbe, Bavarian Danube, Austrian Danube, Mur & Drau).

Correlations between pairwise geographic (GGD) and genetic (*F*_ST_) distances were examined using the Mantel test implemented in the GeneAlEx 6 software [54]. To assess whether significant drainage-wide demographic events may affect our global inferences we tested for both expansion and bottlenecks across groups of populations. Demographic expansion within drainages was assessed using the within-locus k test and the inter-locus g test [55] using the Excel (Microsoft) Macro developed by [56]. The significance of k was evaluated with a one-tailed binomial distribution, whereas the significance of g was based on cut-off values as described in [57].

Finally, to evaluate the potential that genotypic profiles within the Austrian Danube have a hybrid origin, we combined a series of simulated hybrid data sets based on hypothetical hybrid scenarios with Bayesian assessment of membership of individual multi-locus genotypes using the software NewHybrids Version 1.1 [58]. Genotypes from the Austrian Danube basin were tested for membership to various hybrid classes using six different hybrid scenarios, whereby at least one parental source of Atlantic origin was used in five of six scenarios and Atlantic lineage genotypes of both natural and hatchery origin were considered separately. Considering the historical complexity of these systems as well as the size of the data set, our goal with this analysis was to gain global insight on the *plausibility* of various hybrid scenarios without elaborating on the statistical confidence of individual genotype assignment or hybrid architecture of individual populations. As the precise procedure was based to some extent on other major results of our study, more detailed description of these analyses is integrated into the Results section.

## Availability of supporting data

The data sets supporting the results of this article are included within the article and its additional files.

## Competing interests

The authors declare that they have no competing interests.

## Authors’ contributions

EL-K carried out the majority of the laboratory work as well as nearly all statistical analyses and contributed to the first draft of the manuscript as a post-doc employed in the research group of SW. US contributed to the design and development of this research study as well as to the collection and maintenance of all Bavarian sample material. TK contributed substantially to the laboratory work and analysis. SW conceived the design of the project and supervised the research study at all stages, contributed some statistical analyses and designed and wrote the first draft of the manuscript. All authors read, contributed revisions and approved the final manuscript.

## Supplementary Material

Additional file 1 **Population Genetic Statistics of all Populations.** Population genetic statistics for all sampled populations. Shown is the full name of the population, a three letter code, A_R_ = Allelic Richness, *H*_E_ = Expected Heterozygosity, *H*_O_ = Observed Heterozygosity, *F*_*IS*_ = Inbreeding Coefficient, Sig. = P-value for the inbreeding coefficient, whereby *NS* is not significant. Click here for file

Additional file 2 **Additional PCA factors.** Scatterplots of PCA factor 1 (X Axis) against factors 3 through 6 (Y Axis), based on a PCA of microsatellite allele frequencies in all 97 newly typed populations. The additional factors further support several outlier populations as well as the uniqueness of several Pure Danubian populations (e.g. ANR and BIS) and the differentiation between these and Bavarian populations. Several of these populations defining the Y – axis are labeled with the population code for reference. There is however no general pattern or inference that can be drawn from these additional vectors of variation. Click here for file

Additional file 3 **Q-Values of Pure Danubian Populations.** Percentage Self Assignment (Q-values from STRUCTURE analysis) of all pure Danubian populations found in this study. One known stock transport from Anrasersee to the Anlaufbach/Winbach drainage was made and is clearly evidenced here. Additionally, fish from these streams were released into Fuscher Ache by local authorities. Click here for file

Additional file 4 **Complete set of microsatellite data.** Individual allelic data of all microsatellite loci across all populations analyzed in this study. Click here for file
