# Supplementary material for: Genetic variation in brown trout Salmo trutta across the Danube, Rhine, and Elbe headwaters: a failure of the phylogeographic paradigm?
Source: BMC Evol Biol. 2013 Aug 26;13:176. doi: 10.1186/1471-2148-13-176 (PMC3765949; doi:10.1186/1471-2148-13-176)
Supplement: Additional file 1 — Population Genetic Statistics of all Populations. Population genetic statistics for all sampled populations. Shown is the full name of the population, a three letter code, AR = Allelic Richness, HE = Expected Heterozygosity, HO = Observed Heterozygosity, FIS = Inbreeding Coefficient, Sig. = P-value for the inbreeding coefficient, whereby NS is not significant. [file 1471-2148-13-176-S1.docx]

**Additional File 1**. Summary population genetic statistics for the 97 populations typed in this study. Shown are Allelic Richness (A_R_), Expected and Observed Heterozygosities (*H*_E_ & *H*_O,_ respectively), and tests for HWE in terms of *F*_IS_ – values and their corresponding significance values. *NS* = not significant at alpha = 0.05, whereby no table-wide correction is undertaken.

| **Population** | **Pop_Code** | **A_R_** | ***H*_E_** | ***H*_O_** | ***F*_IS_** | **Sig.** |
| --- | --- | --- | --- | --- | --- | --- |
| ***Bavarian Rhine*** | | | | | | |
| Grumpelbach | GRU | 5.1 | 0.701 | 0.654 | 0.100 | *P* = 0.009 |
| Langenaubach | LAN | 4.5 | 0.636 | 0.640 | 0.021 | *NS* |
| Mathelbach | MAT | 6.0 | 0.727 | 0.724 | 0.022 | *NS* |
| Schulmühlbach | SHU | 5.7 | 0.702 | 0.705 | 0.016 | *NS* |
| Thiemitz | THI | 4.3 | 0.616 | 0.592 | 0.068 | *NS* |
| Weisser | WEM | 7.0 | 0.784 | 0.796 | 0.007 | *NS* |
| ***Bavarian Elbe*** | | | | | | |
| Bibersbach | BIB | 6.2 | 0.745 | 0.743 | 0.028 | *NS* |
| Goldbach | GOL | 4.8 | 0.683 | 0.708 | -0.013 | *NS* |
| Harlandtbach | HAL | 5.6 | 0.723 | 0.742 | 0.004 | *NS* |
| Lamitz | LAM | 6.9 | 0.775 | 0.766 | 0.035 | *NS* |
| Steinselb | STB | 6.7 | 0.763 | 0.720 | 0.081 | *P* = 0.0039 |
| Schwarzbach | SWA | 4.7 | 0.685 | 0.660 | 0.064 | *NS* |
| ***Bavarian Danube*** | | | | | | |
| Forellenbach | FOR | 5.1 | 0.626 | 0.639 | 0.006 | *NS* |
| Geiselmoosbach | GEI | 6.2 | 0.748 | 0.749 | 0.02 | *NS* |
| Haarauer Saige | HAA | 4.2 | 0.636 | 0.698 | -0.078 | *P* = 0.0147 |
| Haidenaab | HAD | 5.5 | 0.728 | 0.749 | -0.007 | *NS* |
| Kirnbach | KIR | 6.5 | 0.762 | 0.768 | 0.02 | *NS* |
| Kohlgraben | KOH | 6.5 | 0.757 | 0.786 | -0.015 | *NS* |
| Milchelbach | MIC | 7.0 | 0.776 | 0.770 | 0.032 | *NS* |
| Muhlbach | MUH | 5.7 | 0.700 | 0.708 | 0.011 | *NS* |
| Schlittbach | SLI | 5.7 | 0.722 | 0.756 | -0.021 | *NS* |
| Steingraben | STG | 5.1 | 0.688 | 0.736 | -0.047 | *NS* |
| Tauritzbach | TAU | 6.5 | 0.774 | 0.753 | 0.049 | *P* = 0.0412 |
| Wielenbach | WIE | 5.7 | 0.744 | 0.792 | -0.041 | *NS* |
| ***Austrian Raab*** | | | | | | |
| Hollerbach | HOL | 6.0 | 0.757 | 0.773 | -0,006 | *NS* |
| Raab | RAA | 6.6 | 0.784 | 0.798 | 0 | *NS* |
| ***Austrian Danube*** | | | | | | |
| Aschbach | ASC | 7.1 | 0.803 | 0.823 | -0.009 | *NS* |
| Eidechselbach | EID | 6.4 | 0.784 | 0.822 | -0.031 | *NS* |
| Erlauf | ERL | 6.7 | 0.774 | 0.783 | 0.011 | *NS* |
| Fugnitz | FUG | 6.1 | 0.746 | 0.744 | 0.021 | *NS* |
| Galgenbach | GAL | 4.3 | 0.629 | 0.702 | -0.089 | *P* = 0.0156 |
| Grosse Krems | GKR | 6.4 | 0.773 | 0.761 | 0.029 | *NS* |
| Höllbach | HB | 6.9 | 0.785 | 0.777 | 0.029 | *NS* |
| Hummelmühlbach | HM | 4.6 | 0.604 | 0.600 | 0.039 | *NS* |
| Johnsbach | JOH | 7.3 | 0.808 | 0.816 | 0.005 | *NS* |
| KaYesbach | KAJ | 4.6 | 0.695 | 0.769 | -0.085 | *P* = 0.0096 |
| Kaltergang | KAG | 6.9 | 0.793 | 0.793 | 0.017 | *NS* |
| Kleine Mühl | KM | 6.6 | 0.778 | 0.836 | -0.039 | *NS* |
| **Population** | **Pop_Code** | **A_R_** | ***H*_E_** | ***H*_O_** | ***F_IS_*** | **Sig.** |
| Kleine Ysper | KY | 7.0 | 0.793 | 0.839 | -0.032 | *NS* |
| Kleiner Kamp | KK | 5.6 | 0.743 | 0.728 | 0.024 | *NS* |
| Lohnbach | LOH | 6.0 | 0.729 | 0.757 | -0.02 | *NS* |
| Luckenbach | LUC | 5.6 | 0.721 | 0.818 | -0.081 | *NS* |
| Natters | NAT | 7.2 | 0.816 | 0.847 | -0.026 | *NS* |
| Niklbach | NIK | 5.4 | 0.652 | 0.673 | -0.013 | *NS* |
| Ois | OIS | 7.7 | 0.820 | 0.803 | 0.03 | *P* = 0.0275 |
| Pielach | PIL | 7.5 | 0.815 | 0.798 | 0.036 | *P* = 0.0459 |
| Piesting at Wöll. | PIW | 7.6 | 0.817 | 0.772 | 0.076 | *P* = 0.0021 |
| Preinerbach | PB | 6.8 | 0.791 | 0.739 | 0.083 | *P* = 0.0007 |
| Schwarza | GRA | 7.6 | 0.812 | 0.741 | 0.109 | *P* < 0.001 |
| Thannergraben | THA | 3.4 | 0.585 | 0.558 | 0.063 | *NS* |
| Triebenbach | TRI | 7.2 | 0.792 | 0.774 | 0.039 | *P* = 0.044 |
| Viehbach | VIE | 5.5 | 0.690 | 0.676 | 0.046 | *NS* |
| Weißenbach | WEB | 7.4 | 0.806 | 0.833 | -0.013 | *NS* |
| Weiten | WEI | 7.1 | 0.810 | 0.802 | 0.023 | *NS* |
| ***Austrian Drau*** | | | | | | |
| Anrasersee | ANR | 2.8 | 0.417 | 0.457 | -0.067 | *NS* |
| Bach in der Schütt | BIS | 2.5 | 0.498 | 0.467 | 0.095 | *NS* |
| Elbach | ELB | 6.1 | 0.767 | 0.781 | 0.004 | *NS* |
| Gesgerbach | GES | 5.4 | 0.709 | 0.780 | -0.076 | *P* = 0.0173 |
| Gößbach | GÖß | 6.9 | 0.784 | 0.813 | -0.011 | NS |
| Lichtengrabenbach | LIC | 6.0 | 0.694 | 0.670 | 0.056 | *P* = 0.0341 |
| Oberallacher Bach | OBE | 5.4 | 0.689 | 0.731 | -0.029 | NS |
| Radlbach | RAD | 6.6 | 0.715 | 0.699 | 0.048 | NS |
| Rakoutzabach | RAK | 6.1 | 0.741 | 0.782 | 0.004 | NS |
| Saggrabenbach | SAG | 4.3 | 0.561 | 0.616 | -0.043 | NS |
| Schafgrabenbach | SCH | 4.8 | 0.573 | 0.571 | 0.029 | NS |
| Stieger Wiesenbach | SWB | 4.8 | 0.585 | 0.567 | 0.064 | NS |
| Stutterner Bach | STU | 5.1 | 0.679 | 0.638 | 0.101 | *P* = 0.024 |
| Wellenbach | WEL | 5.5 | 0.714 | 0.763 | -0.034 | *NS* |
| Wolfsgrabenbach | WOL | 3.1 | 0.480 | 0.497 | -0.005 | *NS* |
| ***Austrian Mur*** | | | | | | |
| Etrachbach | ETR | 3.0 | 0.403 | 0.381 | 0.071 | *NS* |
| Kainach | KAI | 7.8 | 0.831 | 0.857 | -0.012 | *NS* |
| Kotalmbach | KOT | 3.9 | 0.506 | 0.470 | 0.09 | *P* = 0.0081 |
| Lassnitz | LAS | 6.1 | 0.731 | 0.723 | 0.028 | *NS* |
| Modriachwinkelbach | MOD | 6.9 | 0.767 | 0.773 | 0.007 | *NS* |
| Moosbach | MOO | 2.6 | 0.374 | 0.398 | -0.047 | *NS* |
| Mürz (Feistritz) | MÜR | 6.7 | 0.779 | 0.822 | -0.038 | *NS* |
| Mürz | MRZ | 6.7 | 0.779 | 0.778 | 0.018 | *NS* |
| Rosenbach | ROS | 4.8 | 0.697 | 0.641 | 0.097 | *P* = 0.0009 |
| Schwarze Sulm | SUL | 7.9 | 0.836 | 0.854 | -0.008 | *NS* |
| Steinbach | STE | 5.3 | 0.685 | 0.710 | -0.016 | *NS* |
| Teigitsch | TEI | 6.6 | 0.772 | 0.801 | -0.024 | *NS* |
| Tiefbach | TIE | 5.6 | 0.658 | 0.677 | -0.008 | *NS* |
| Wiesenriegelbach | WRB | 6.1 | 0.750 | 0.730 | 0.054 | *NS* |
| **Population** | **Pop_Code** | **A_R_** | ***H*_E_** | ***H*_O_** | ***F_IS_*** | **Sig.** |
| ***Austrian Inn*** | | | | | | |
| Anlaufbach | ANL | 3.4 | 0.598 | 0.529 | 0.156 | *P =* 0.007 |
| Winbach | WIS | 4.2 | 0.642 | 0.685 | 0.118 | *P =* 0.0003 |
| Fuscher | FUS | 3.3 | 0.554 | 0.501 | 0.110 | *P =* 0.0006 |
| Blühnbach | BLU | 6.1 | 0.675 | 0.702 | -0.022 | *NS* |
| ***Hatcheries*** | | | | | | |
| Hatchery | A | 6.9 | 0.800 | 0.823 | -0.011 | *NS* |
| Hatchery | B | 7.0 | 0.801 | 0.826 | -0.014 | *NS* |
| Hatchery | C | 7.3 | 0.816 | 0.799 | 0.043 | *NS* |
| Hatchery | D | 5.0 | 0.709 | 0.762 | -0.059 | *P =* 0.0256 |
| Hatchery | E | 6.0 | 0.737 | 0.820 | -0.094 | *P =* 0.0002 |
| Hatchery | F | 5.9 | 0.756 | 0.747 | 0.024 | *NS* |
| Hatchery | G | 6.8 | 0.775 | 0.794 | -0.007 | *NS* |
| Hatchery | H | 6.8 | 0.788 | 0.781 | 0.03 | *NS* |
| Hatchery | I | 6.1 | 0.766 | 0.802 | -0.028 | NS |
| Hatchery | J | 3.9 | 0.610 | 0.683 | -0.103 | 0.0029 |
