# Supplementary material for: Genetic variation in brown trout Salmo trutta across the Danube, Rhine, and Elbe headwaters: a failure of the phylogeographic paradigm?
Source: BMC Evol Biol. 2013 Aug 26;13:176. doi: 10.1186/1471-2148-13-176 (PMC3765949; doi:10.1186/1471-2148-13-176)
Supplement: Additional file 2 — Additional PCA factors. Scatterplots of PCA factor 1 (X Axis) against factors 3 through 6 (Y Axis), based on a PCA of microsatellite allele frequencies in all 97 newly typed populations. The additional factors further support several outlier populations as well as the uniqueness of several Pure Danubian populations (e.g. ANR and BIS) and the differentiation between these and Bavarian populations. Several of these populations defining the Y – axis are labeled with the population code for reference. There is however no general pattern or inference that can be drawn from these additional vectors of variation. [file 1471-2148-13-176-S2.docx]

**Additional File 2**. Scatterplots of PCA factor 1 (X Axis) against factors 3 through 6 (Y Axis), based on a PCA of microsatellite allele frequencies in all 97 newly typed populations. The additional factors further support several outlier populations as well as the uniqueness of several Pure Danubian populations (e.g ANR and BIS) and the differentiation between these and Bavarian populations. Several of these populations defining the Y – axis are labeled with the population code for reference. There is however no general pattern or inference that can be drawn from these additional vectors of variation.


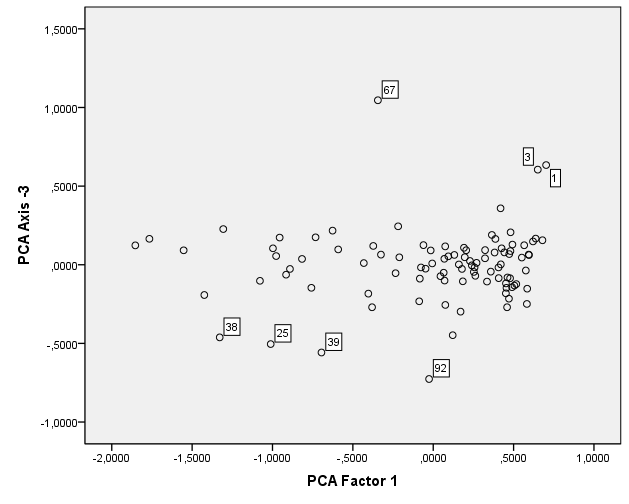


**WOL**

**BIS**

**ANR**

**HM**

**THA**

**HAA**

**FOR**


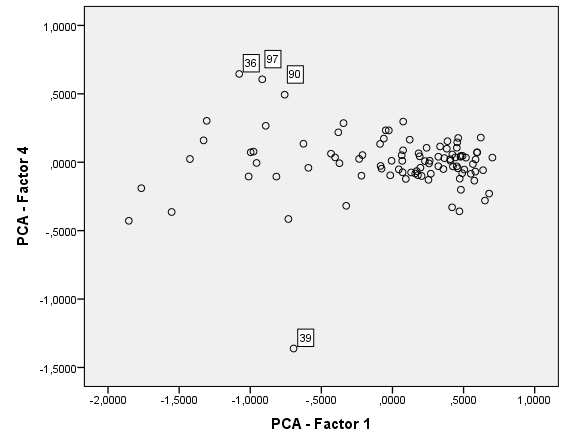


**SWB**

**NIK**

**FUS**

**ANR**


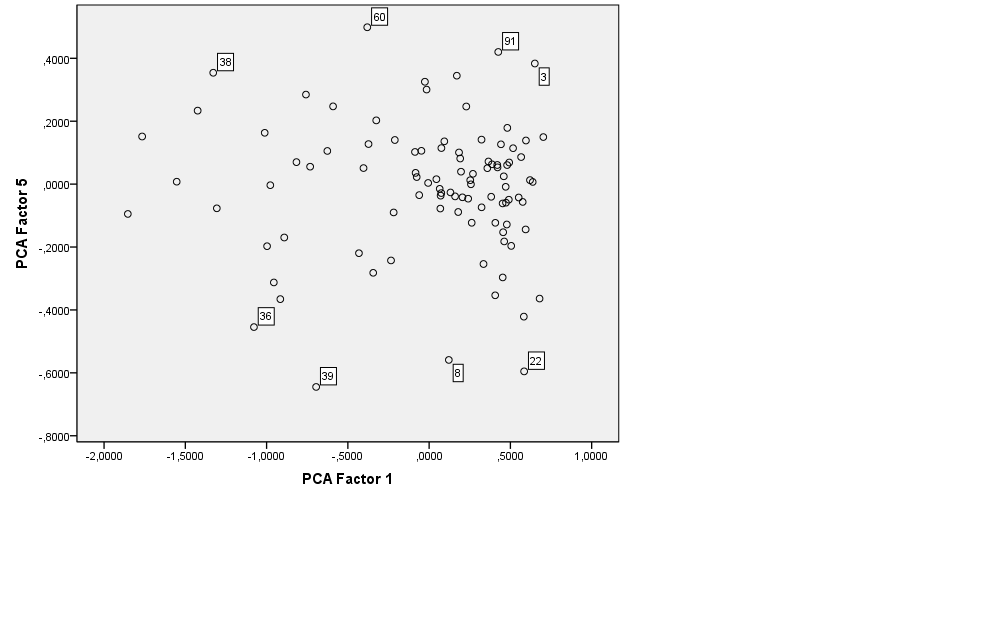


**SHU**

**MUH**

**ANR**

**SWB**

**HAA**

**GAL**

**LOH**

**WOL**


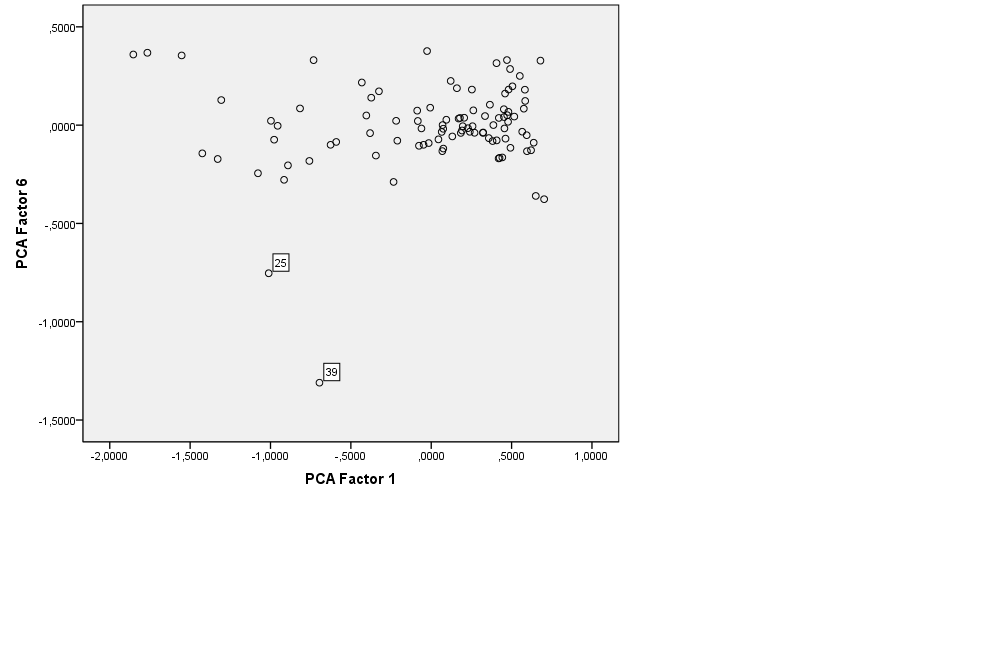


**BIS**

**ANR**
