# Supplementary material for: Genetic variation in brown trout Salmo trutta across the Danube, Rhine, and Elbe headwaters: a failure of the phylogeographic paradigm?
Source: BMC Evol Biol. 2013 Aug 26;13:176. doi: 10.1186/1471-2148-13-176 (PMC3765949; doi:10.1186/1471-2148-13-176)

Additional File_2. Percentage Self Assignment (Q-values from STRUCTURE analysis) of all pure Danubian populations found in this study. One known stock transport from Anrasersee to the Anlaufbach/Winbach drainage was made and is clearly evidenced here. Additionally, fish from these streams were released into Fuscher Ache by local authorities

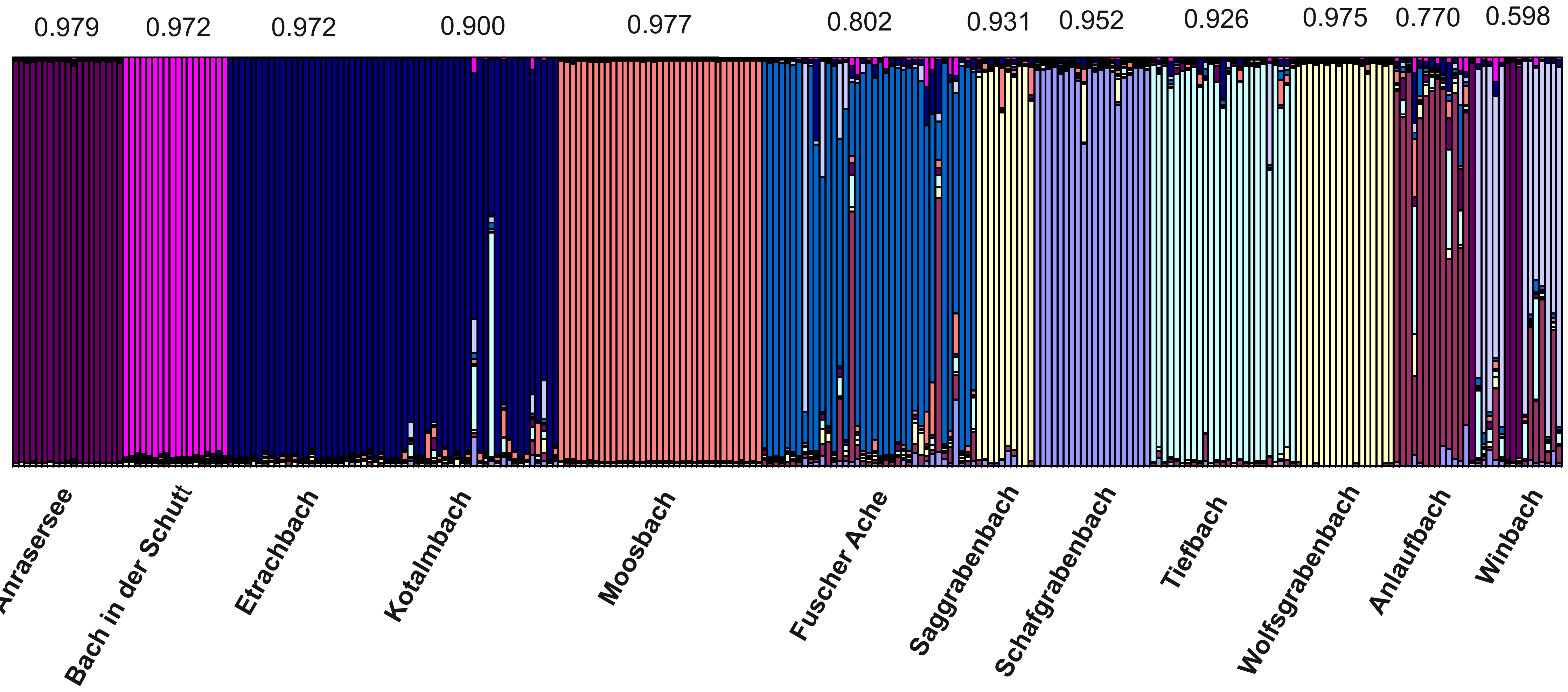

Supplement: Additional file 3 — Q-Values of Pure Danubian Populations. Percentage Self Assignment (Q-values from STRUCTURE analysis) of all pure Danubian populations found in this study. One known stock transport from Anrasersee to the Anlaufbach/Winbach drainage was made and is clearly evidenced here. Additionally, fish from these streams were released into Fuscher Ache by local authorities. [file 1471-2148-13-176-S3.pdf]
