# Supplementary material for: Genetic variation in brown trout Salmo trutta across the Danube, Rhine, and Elbe headwaters: a failure of the phylogeographic paradigm?
Source: BMC Evol Biol. 2013 Aug 26;13:176. doi: 10.1186/1471-2148-13-176 (PMC3765949; doi:10.1186/1471-2148-13-176)

Additional File_3. Percentage Self Assignment (Q-values from STRUCTURE analysis) of all pure Danubian populations found in this study. One known stock transport from Anrasersee to the Anlaufbach/Winbach drainage was made and is clearly evidenced here. Additionally, fish from these streams were released into Fuscher Ache by local authorities.

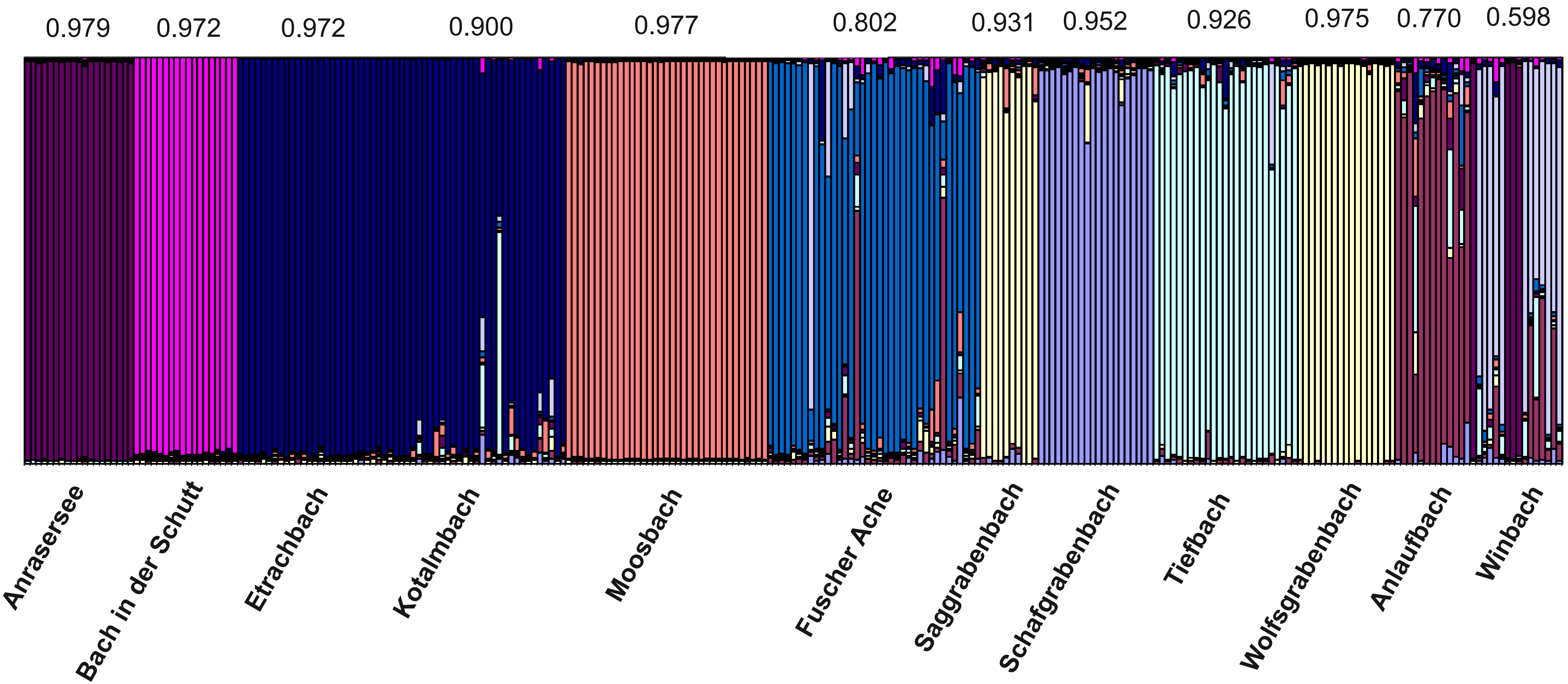

Supplement: Additional file 4 — Complete set of microsatellite data. Individual allelic data of all microsatellite loci across all populations analyzed in this study. [file 1471-2148-13-176-S4.pdf]
